# Barriers to care among people with schizophrenia attending a tertiary psychiatric hospital in Nigeria

**DOI:** 10.4102/sajpsychiatry.v25i0.1392

**Published:** 2019-10-21

**Authors:** Bawo O. James, Felicia I. Thomas, Omonefe J. Seb-Akahomen, Nosa G. Igbinomwanhia, Chinwe F. Inogbo, Graham Thornicroft

**Affiliations:** 1 Department of Clinical Services, Federal Neuro-Psychiatric Hospital, Benin City, Nigeria; 2 Synapse Centre for Psychological Medicine, Lagos, Nigeria; 3 Centre for Global Mental Health, Institute of Psychiatry, Psychology and Neurosciences, Kings College London, England, United Kingdom

**Keywords:** barriers to care, schizophrenia, stigma, Nigeria, attitudes

## Abstract

**Background:**

Individuals with schizophrenia in low- and middle-income countries and their caregivers face multiple barriers to care-seeking and continuous engagement with treatment services. Identifying specific barrier patterns would aid targeted interventions aimed at improving treatment access.

**Aim:**

The aim of this study was to determine stigma- and non-stigma-related barriers to care-seeking among persons with schizophrenia in Nigeria.

**Setting:**

This study was conducted at the Outpatient Clinics of the Federal Neuro-Psychiatric Hospital, Benin City, Nigeria.

**Methods:**

A cross-sectional study of a dyad of persons with schizophrenia and caregivers (*n* = 161) attending outpatient services at a neuro-psychiatric hospital in Nigeria. Stigma- and non-stigma-related barriers were assessed using the 30-item Barriers to Access to Care Evaluation (BACE) scale.

**Results:**

Lack of insight, preference for alternative care, illness severity and financial constraints were common barriers to care-seeking among persons with schizophrenia. Females were significantly more likely to report greater overall treatment barrier (*p* < 0.01) and stigma-related barriers (*p* < 0.02).

**Conclusion:**

This study shows that attitudinal barriers impede care access and engagement among persons with schizophrenia in Nigeria.

## Background

Over 21 million people globally live with schizophrenia, a disabling mental disorder, affecting approximately 4–5 million individuals living in African countries.^1^ In Nigeria, pathways to care for mental disorders are complex and significantly influenced by stigma, poor understanding about these illnesses, cultural and religious factors, financial constraints, and poor integration of mental healthcare into primary healthcare systems.^2,3^ Therefore, only a minority of persons with mental illness access appropriate care in a timely manner. Furthermore, constant engagement with appropriate mental healthcare is low, even in tertiary hospital settings that provide the highest level of clinical expertise.^4,5^

Although some research has been conducted on pathways to care for mental disorders in Nigeria,^6,7^ there is a lack of evidence for the specific barriers (from the perspective of patients with schizophrenia and their caregivers) that prevent or discourage them from accessing mental health services or continuing with services after initial engagement. In the United Kingdom, stigma-related barriers (relating to employability and parenting roles) were major barriers in a cohort receiving care for mental health disorders. Professionals in mental healthcare also constitute a barrier to care albeit inadvertently. In addition, in developing countries, income-related disparities also serve as major non-stigma barriers to healthcare access.^8,9,10^

The aim of this study was to determine the barriers to access to mental healthcare among patients with schizophrenia and their caregivers attending a tertiary mental healthcare service in southern Nigeria. We specifically sought to identify barriers to mental healthcare for patients with schizophrenia and their caregivers and explore socio-demographic correlates.

## Methods

### Study design

This was a cross-sectional descriptive study.

### Study location

This study was conducted at the Federal Neuro-Psychiatric Hospital, Benin City. This is a tertiary healthcare facility funded by the Federal Government of Nigeria, which provides inpatient and outpatient mental health services, and community extension services.

### Study participants

The study participants were adult patients aged 18–64 years with a diagnosis of schizophrenia who had been attending the outpatient clinics for at least 6 months. Secondly, caregivers of adult patients with a diagnosis of schizophrenia who had been attending the outpatient clinic for at least 3 months were also interviewed. We excluded dyads who declined consent to participate in the study, patients who could not take a decision to participate in the study or were too ill to be interviewed.

### Operational definition of ‘caregiver’

For the purpose of this study, a caregiver was defined as a first-degree relative and a non-professional, non-paid person who was mostly involved with the everyday care of a patient. He or she would also be most likely to respond to any request for special assistance at any time, by the patient.^11^

### Sample size

The participants consisted of consecutive sample of 161 patients and 161 caregivers who met the criteria for inclusion in the study, attending the hospital between April and September 2018.

### Study instruments

- *A semi-structured questionnaire designed by the researchers* included information about gender, age, religion, level of formal education, duration of illness, duration of treatment and beliefs about aetiology of schizophrenia.
- *The Barriers to Care Evaluation (BACE) version 4*: This scale was developed and validated at the Health Services and Population Research Department, Institute of Psychiatry, King’s College London, to assess barriers to mental healthcare for people with mental health problems. Its psychometric properties have been established and it has good reliability and validity.^9^ It is a 30-item scale that can be self-administered or interviewer-administered. The response to each question is rated on a Likert scale of 0 (not at all) to 3 (a lot), with higher scores indicating greater barrier. The overall BACE score (mean of rating for all applicable items) may be used. Three different scores may be given for each barrier:
  ■ mean of the response scores
  ■ percentage reporting having experienced the barrier to any degree
  ■ percentage that rated the barrier as a major barrier (rating of 3).

The BACE treatment stigma subscale score is the mean of stigma-related items (3, 5, 8, 9, 12, 14, 17, 19, 21, 24, 26 and 28). In a similar way, subscale scores for instrumental barriers (items 1, 6, 11, 15, 16, 27, 29 and 30) and attitudinal barriers (items 2, 4, 7, 10, 13, 18, 20, 22, 23 and 25) may be calculated.

### Procedure

All the interviewers (three psychiatrists) attended a start-up meeting, during which they were guided on how to complete the BACE questionnaire. The participants were interviewed in the private consulting rooms of the outpatient clinic. The interviews went thus: the semi-structured questionnaire was administered to the caregivers and to the patients, while the BACE questionnaire was administered to the patients alone (although with the caregivers in attendance). The case files of all the patients who participated in the study were tagged to prevent interviewing the same patient more than once.

### Data analysis

Data were summarised using means and standard deviations, and displayed in tables. Reliability of the overall and stigma subscale of the BACE was determined using the Cronbach’s alpha. The association between continuous variables was determined using *t*-test. Level of significance was set at *p < 0.05.*

### Ethical considerations

Study protocol was approved by the Ethics and Research Committee of the Federal Neuro-Psychiatric Hospital, Benin City, Nigeria.

Ethical approval for this study was obtained from the Ethics Review Committee of the Federal Neuro-Psychiatric Hospital, Benin City. Participants were informed about the nature and aim of the study, and verbal informed consent was obtained. Confidentiality and anonymity were maintained. Participants were also informed that they were free to opt out of the study at any time if they so desired, with no negative consequences as regards their treatment.

## Results

### Socio-demographic characteristics of patients and caregivers

The average age (standard deviartion [s.d.]) of patients in this sample was 36.5 (9.2) years, with most in the 31–40 years’ age range.

There were slightly more males (*n* = 84; 52.2%) and most were Christian (*n* = 155; 96.3%). Over half had a secondary level of education (*n* = 95; 59.0%). A majority were single (*n* = 119; 73.9%), unemployed (*n* = 97; 60.2%) and living with a family member (*n* = 132; 81.3%) and not caring for a minor (*n* = 125; 77.6%) (see Table 1).

**TABLE 1:** Socio-demographic characteristics of patients.

Variable	Frequency	Percentage
**Age (years)**		
18–30	47	29.2
31–40	73	45.3
41–50	24	14.9
51–65	17	10.5
**Mean age (s.d.)**	36.5 (9.2)	-
**Gender**		
Male	84	52.2
Female	77	47.8
**Religion**		
Christianity	155	96.3
Islam	5	3.1
ATR	1	0.6
**Level of education**		
Primary	38	23.6
Secondary	95	59.0
Tertiary	28	17.4
**Employed**		
Yes	57	35.4
No	97	60.2
Retired	3	1.9
Student	4	2.5
**Marital status**		
Single	119	73.9
Married	29	18.0
Widowed	3	1.9
Separated	10	6.2
**Living**		
Alone	3	1.9
With parent	102	62.7
With sibling(s)	30	18.6
With others	25	26.8
**Caring for a minor**		
Yes	36	22.4
No	125	77.6

ATR, African Traditional Religion; s.d., standard deviation.

Average caregiver age was 52.8 (13.3) years, and most were female (*n* = 112; 69.6%). A majority were Christians (*n* = 153; 95%) and employed (*n* = 122; 75.8%). Slightly under half of caregivers rated their ‘ease of bringing their relatives to hospital’ as ‘difficult/very difficult’ (*n* = 77; 47.9%). Over half of caregivers were parents (*n* = 85; 52.8%), as shown in Table 2.

**TABLE 2:** Socio-demographic characteristics of caregivers.

Variable	Frequency	Percentage
**Age (years)**		
18–45	42	26.1
46–55	46	28.6
56–65	42	26.1
66–80	31	19.3
**Mean age (s.d.)**	52.8 (13.3)	-
**Gender**		
Male	49	30.4
Female	112	69.6
**Religion**		
Christianity	153	95.0
Islam	4	2.5
ATR	4	2.5
**Level of education**		
No formal education	10	6.2
Primary	51	31.7
Secondary	60	37.3
Tertiary	40	24.8
**Employment status**		
Employed	122	75.8
Unemployed	20	12.4
Retired	17	10.6
Student	2	1.2
**Marital status**		
Single	20	12.4
Married/cohabiting	95	59.0
Divorced/separated	8	4.9
Widowed	38	23.6
**Ease of coming to hospital?**		
Very easy	18	11.2
Easy	66	41.0
Difficult	65	40.4
Very difficult	12	7.5
**Relationship to patient**		
Parent	85	52.8
Sibling	45	28.0
Aunt/Uncle	4	2.5
Spouse	13	8.1
Other	14	8.7

ATR, African Traditional Religion; s.d., standard deviation.

### Barriers to care

The reliability of the overall BACE scale was 0.80, while that of the 12-item treatment stigma subscale was 0.87. The stigma-related barriers most commonly endorsed (identified as a major barrier) were ‘feeling embarrassed or ashamed’ (*n* = 20; 12.4%), ‘concern that I might be seen as crazy’ (*n* = 15; 9.3%) and ‘concern about what my friends might think, say or do’ (*n* = 12; 7.5%) (see Table 3).

**TABLE 3:** Stigma-related barriers.

Stigma-related barrier items	Reporting item as a barrier to any degree		Reporting item as a major barrier		Mean	s.d.
	*n*	%	*n*	%		
Feeling embarrassed or ashamed	78	48.4	20	12.4	0.86	1.06
Concern that I might be seen as ‘crazy’	82	50.9	15	9.3	0.85	1.00
Concern that people I know might find out	73	45.3	12	7.5	0.75	0.96
Concern about what my friends might think, say or do	79	49.1	12	7.5	0.80	0.97
Concern that people might not take me seriously if they found out I was having professional care	62	38.5	11	6.8	0.68	0.97
Concern about what my family might think, say, do or feel	27	16.8	9	5.6	0.34	0.83
Not wanting a mental health problem to be on my medical records	36	22.4	8	5.0	0.37	0.80
Concern that it might harm my chances when applying for jobs	22	13.7	6	3.7	2.8	0.75
Concern about what people at work might think, say or do	18	11.2	4	2.5	0.20	0.62
Concern that I might be seen as weak for having a mental health problem	25	15.5	3	1.9	0.25	0.66
Concern that I might be seen as a bad parent	9	5.6	1	0.6	0.09	0.39
Concern that my children may be taken into care or that I may lose access or custody without my agreement	1	0.6	1	0.6	0.02	0.24

s.d., standard deviartion.

Table 4 shows that common attitudinal barriers endorsed were ‘thinking that I do not have a problem’ (*n* = 60; 37.3%), ‘preferring to get alternative forms of care’ (*n* = 33; 20.5%) and ‘wanting to solve the problem on my own’ (*n* = 23, 14.3%).

**TABLE 4:** Attitudinal barriers.

Attitudinal barrier items	Reporting item as a barrier to any degree		Reporting item as a major barrier		Mean	s.d.
	*n*	%	*n*	%		
Thinking I did not have a problem	132	82	60	37.3	1.88	1.11
Preferring to get alternative forms of care (e.g. traditional/religious healing or alternative/complementary therapies)	106	65.8	33	20.5	1.28	1.14
Wanting to solve the problem on my own	101	62.7	23	14.3	1.20	1.10
Thinking the problem would get better by itself	91	56.5	11	6.8	0.97	0.99
Concerns about the treatments available (e.g. medication side effects)	69	42.9	10	6.2	0.75	0.98
Fear of being put in hospital against my will	30	18.6	6	3.7	0.34	0.78
Thinking that professional care probably would not help	44	27.8	5	3.1	0.42	0.77
Dislike of talking about my feelings, emotions or thoughts	20	12.4	2	1.2	0.19	0.57
Preferring to get help from family or friends	15	9.3	1	0.6	0.12	0.41
Having had previous bad experiences with professional care for mental health	8	5.0	0	0	0.08	0.37

s.d., standard deviartion.

Findings highlighted in Table 5 reveal that instrumental barriers commonly endorsed were ‘being too unwell to ask for help’ (*n* = 38; 23.6%), ‘problems with transport or travelling to appointments’ (*n* = 30; 18.6%) and ‘financial constraints and unsure where to get professional help’ (*n* = 28, 17.4%).

**TABLE 5:** Instrumental barriers.

Instrumental barrier items	Reporting item as a barrier to any degree		Reporting item as a major barrier		Mean	s.d.
	*n*	%	*n*	%		
Being too unwell to ask for help	124	77	38	23.6	1.48	1.09
Problems with transport or travelling to appointments	77	47.8	30	18.6	1.04	1.21
Not being able to afford the financial costs involved	101	62.7	28	17.4	1.91	1.12
Being unsure where to go to get professional care	107	66.5	28	17.4	1.25	1.10
Difficulty taking time off work	8	5	1	0.6	0.07	0.34
Having problems with childcare while I receive professional care	2	1.2	1	0.6	0.03	0.28
Having no one who could help me get professional care	1	0.6	0	0	0.01	0.16
Professionals from my own ethnic or cultural group not being available	3	1.9	0	0	0.02	0.19

s.d., standard deviartion.

### Gender differences

There were differences in reported barriers across gender. Female patients were significantly more likely to report barriers overall compared with males (19.91 [11.30] vs. 15.92 [7.55], *t* = 2.70, *p* < 0.01). A similar pattern was observed in the stigma subscale (6.65 [6.87] vs. 4.43 [4.97], *t* = 2.36, *p* < 0.02). No significant differences were seen when instrumental barriers (*p* = 0.24) and attitudinal barriers (*p* = 0.05) were compared.

## Discussion

This study showed that barriers to care are still common among persons with schizophrenia receiving care in a low- and middle-income country. Specifically, a lack of insight into the illness continues to hamper constant engagement with available treatment services. The ignorance about the illness and its course is further amplified when participants endorsed seeking alternative forms of care as a barrier to effective treatment. Consequently, we infer that these attitudinal barriers may have resulted in prolonged duration of untreated psychosis. Expectedly, financial difficulties and problems with transport logistics were also identified as major instrumental barriers. In Nigeria, payment for mental healthcare is largely borne by patients or their family members, with a minority having access to limited health insurance. We also report that stigma as a barrier to care had a higher deleterious effect on female patients compared with males.

The validation study of the BACE conducted among service users in the United Kingdom^9^ identified that stigma-related barriers were major barriers, whereas attitudinal barriers were dominant in the Nigerian sample. A study utilising the BACE to ascertain health-seeking prospects of young adults in the United Kingdom saw a marginal preponderance of attitudinal barriers compared with stigma-related barriers.^12^ Although earlier reports from Nigeria identify that self-stigma is common among the mentally ill,^13,14^ it seems that its influence as a treatment or care barrier is mediated by the role of caregivers in treatment-seeking. Thus, a dynamic interaction exists between the individuals recognising symptoms as those of a mental illness, recognising the need for treatment and seeking the support of caregivers to access or pay for care.

The finding that lack of insight was a major barrier to seeking care is consistent with beliefs about the aetiology of mental illness. Individuals in Nigeria are more likely to endorse spiritual or magico-religious aetiologies or the role of psychoactive drugs and therefore seek alternative care options that are consistent with their belief systems as regards the aetiology of their symptoms.^15–17^ Often, it is the failure of alternative and complementary systems of care that presents the individual or family members with no other choice than to present to orthodox care services. A recent review identified ‘lack of knowledge and understanding’ as one of the overarching themes that modify pathways to care for persons with mental illness.^18^

The increased likelihood of females reporting a barrier is consistent with a recent robust evaluation conducted in the United Kingdom.^19^ Females in low- and middle-income countries need to overcome cultural practices and attitudinal dispositions in society that reduce empowerment and access to available services. More commonly, symptom severity is greater in females before they can get the support from family to access care.

### Strengths and limitations

This is the first study to report on barriers to care utilising the BACE in a low- and middle-income country. Another strength of the study was its ability to show that gender differences play a role in perceptions of the illness, seeking care and attitudes within a defined sub-culture. We therefore postulate that the knowledge of gender differences in attitudes, illness perception and care-seeking would facilitate the design of interventions aimed at overcoming the barriers to seeking care in patients with schizophrenia. A possible limitation is the fact that participants were drawn from a single hospital setting; however, studies have shown that not much difference exists across the country as regards the belief systems about mental illness, early presentation and seeking alternative treatment options.^20,21^ In addition, the BACE was interviewer-administered, so that problems with literacy and comprehension could be overcome. Also, we noted that the percentage of patients reporting items on the BACE as a major barrier seems low, while percentages reporting items to some degree are fairly high. This might possibly be a limitation of this study that may be accounted for by interviewer bias. Furthermore, the fact that a caregiver had to form part of the study might in itself be seen as an instrumental barrier to care, as some patients may require constant guidance from caregivers, hence the need to accompany them to the hospital for treatment.

### Implications for future research and practice

We note that even among individuals already receiving care in mental health services, the level of stigma- and non-stigma-related barriers is high. It is essential therefore to determine how stigma-related factors impede care access among persons at risk, or with mental health problems but yet to access available services. Secondly, research on interventions that reduce stigma as well as incentives that improve treatment access, particularly among the economically disadvantaged, are needed. There is evidence that the poor usually have a poorer uptake of available and effective treatment services.

Lastly, mental health professionals need to be aware that stigma may still impede treatment engagement even among those who routinely access care. Providing information, identifying and eliminating systematic practices or behaviours that promote stigma will benefit barrier elimination.

## Conclusion

Persons with schizophrenia receiving tertiary level care in Nigeria report several stigma- and non-stigma related barriers to care. Specifically, a lack of insight, a preference for alternative care, greater illness severity and a lack of money to pay for care were commonly endorsed by participants. These barriers were more prevalent among persons with schizophrenia who were female.
